# Optical and Clinical Outcomes of an Isofocal Intraocular Lens vs. a Monofocal Standard Lens

**DOI:** 10.3390/life13102001

**Published:** 2023-09-30

**Authors:** Lidia Pérez-Sanz, Veronica Gonzalez-Fernandez, José Antonio Gómez-Pedrero, César Albarrán-Diego, María García-Montero, Nuria Garzón

**Affiliations:** 1 Optometry and Vision Department, Facultad de Óptica y Optometría, Universidad Complutense de Madrid, C/Arcos de Jalón, 118, 28037 Madrid, Spain; lidiampe@ucm.es (L.P.-S.); mgarc01@ucm.es (M.G.-M.); mgarzonj@ucm.es (N.G.); 2 Miranza IOA, C/Galileo, 104, 28003 Madrid, Spain; 3 Optics Department, Facultad de Óptica y Optometría, Universidad Complutense de Madrid, C/Arcos de Jalón, 118, 28037 Madrid, Spain; veronicagf@ucm.es; 4 Departament d’Òptica i Optometria i Ciències de la Visió, Facultat de Física, Universitat de València, C/Doctor Moliner, 46100 Burjassot, Spain; cesar.albarran@uv.es

**Keywords:** cataract, isofocal, extended depth of focus, intraocular lens

## Abstract

The aim of this study is to evaluate the results obtained on the optical bench and clinically with an isofocal lens (ISOPure, BVI medical, Belgium) to compare them to a standard monofocal one (MicroPure, BVI medical, Belgium). To do so, we have combined laboratory investigation and a prospective, comparative, and randomized clinical study. First, we have measured the wavefront of the two models studied using a NIMO TR1504 (Lambda-X, Belgium) deflectometer for three nominal powers: +10.00, +20.00 and +30.00 D. In the randomized study with 48 patients, half of them implanted with ISOPure and the other with MicroPure, we have measured visual acuities and contrast sensitivity under photopic and mesopic conditions. The optical bench results show that the isofocal lens presented higher power than the monofocal one, at the lens center, due to the spherical aberration (coefficients Z(4,0), Z(6,0) and Z(8,0)) induced by the greater asphericity of its design. The addition obtained depended on the nominal power, from +1.00 to +1.50 D. The results of the clinical study showed that the ISOPure lens presented better visual outcomes, which were statistically significant, at intermediate distance compared to the MicroPure lens (*p*-values of 0.014 and 0.022 for 80 and 60 cm, respectively) without decreasing the contrast sensitivity. Clinical outcomes were not affected by pupillary size. In conclusion, due to the increase in power at the lens center due to its highly aspherical design, the isofocal lens evaluated showed better intermediate vision than the monofocal one.

## 1. Introduction

The great evolution that has been taking place in intraocular lens (IOL) designs in recent years forces us to constantly update our knowledge to be able to identify the advantages or disadvantages that these designs may present for each patient, but this challenge is also what allows us to offer increasingly customized solutions, always in search of the best results [1,2,3]. The latest advances have been primarily focused on designing lenses that offer an increased range of vision, enabling continuous good visual acuity from far to intermediate distances while maintaining good contrast sensitivity and minimizing dysphotopsias.

One of these new designs applied for the first time in IOLs is the isofocal design, with the ISOPure lens manufactured by the BVI-Physiol laboratory (Liège, Belgium).

The ISOPure lens is a bi-aspheric refractive extended-depth-of-focus (EDOF) lens. The lens is designed in conjunction to an eye model, so the lens is optimized to obtain the best results of the visual metrics of the eye model for a range of distances and pupil apertures [4]. The lens presents a rotationally symmetric power distribution, with concentric zones of constant power with smooth power transition between them. The power is higher at the central region of the lens and lower at the periphery according to the patent [4], thus inducing negative spherical aberration. The aspheric surfaces are the combination of a conicoid and a polynomial with even powers widely employed in optics [5]. The inclusion of even aspherical coefficients from the 4th to 10th order in the ISOPure aspheric surfaces significantly improves the multifocal optical performance as described by designers. The inventors refer to the resulting design as isofocal [4].

The available information on this lens through scientific publications is still limited, both at the level of optical bench testing and clinical studies. Bova et al. [6] found better intermediate visual acuity and similar contrast sensitivity results for the isofocal IOL compared to a monofocal control lens. On an optical bench [7], greater pupil dependency in distance image quality has been evidenced. To date, there has been no publication that combines the two types of results.

Therefore, the purpose of this study is to characterize the lens using a commercial optical bench, the NIMO TR1504 system (Lambda-X, Nivelles, Belgium). The study aims to determine the power profiles and spherical aberration (SA) across the entire surface of the lens for three different powers (+10.00, +20.00, and +30.00 D). Subsequently, the objective results obtained will be compared to the subjective results obtained in a clinical study involving patients. Additionally, the obtained results will be compared to those of a control lens, the MicroPure model (BVI-Physiol, Liège, Belgium), which shares the same material and platform design, but with a monofocal optical design.

## 2. Materials and Methods

### 2.1. Optical Bench

The NIMO TR1504 device is a deflectometer that operates based on the phase-shifting Schlieren principle [8] and combines it with the phase-shifting method of interferometry [9].

The measurement operation consists of applying the phase-shifting principle to map the light beam deviation on the camera. The instrument’s resolution depends on the number of pixels in the camera, leading to a higher resolution compared to other methods. Customized software is then used to calculate various power-related dimensions of the lens, such as power, sphere, cylinder, and axis. This is achieved through fitting the calculated wavefront to a Zernike polynomial combination. Additionally, high-resolution power maps are calculated for each pixel within the optic zone of the lens. The instrument’s software also enables wavefront analysis via Zernike polynomial decomposition at different aperture diameters of the lens [10].

Through measuring the fringe pattern distortion using phase-shifting techniques, it is possible to compute the light deflection and hence the wavefront and power [11]. The instrument’s light source exhibits a radiance peak at 546 nm, which is close to the spectral relative luminance efficacy peak of the human visual system located at 555 nm under photopic conditions [12].

In this way, this device can provide information on various characteristics of IOLs, including power profiles and spherical aberration profiles.

The measurement protocol used followed the same procedure as described by Gomez-Pedrero et al. [13] for intraocular lenses. It involved conducting 10 measurements for each evaluation without the use of filters, considering the lenses as thin lenses.

### 2.2. Intraocular Lenses

The ISOPure lens is an extended-depth-of-focus (EDOF) IOL based on patented polynomial technology with bi-aspherical surfaces. It involves certain surface modifications with impact on Zernike coefficients up to the 10th order to create an anterior and posterior surface profile with increased negative spherical aberration from the center to the periphery of the optic. The aim of this design is to extend the depth of focus compared to a monofocal lens [4].

The MicroPure lens is a biconvex aspheric standard monofocal lens. The posterior surface of the lens has a conic profile to correct −0.11 μm of SA to partially compensate for the positive spherical aberration of the average human cornea, without the manufacturer providing information regarding the optical zone related to this value of spherical aberration induction.

Both lenses are made of hydrophobic acrylic material that is glistening-free (G-free). They feature four closed haptics and include an ultraviolet and light blue filter. The total diameter of the lenses is 11.0 mm, while the optic diameter is 6.0 mm. The refractive index of the lenses is 1.53, and the Abbe number is 42.

### 2.3. Clinical Study

A prospective, comparative, randomized study was conducted on patients undergoing cataract surgery with bilateral implantation of the same IOL model in both eyes. The ‘randomizr’ package for RStudio version 4.3.1 was used to automate the random assignment process, so the software assigned each subject to receive either bilateral ISOPure implantation or bilateral MicroPure implantation (https://declaredesign.org/r/randomizr/ accessed on 10 June 2020). Written informed consent was obtained from all patients after they were informed about the nature of the study, protocols, and implications of their participation. The study was approved by the clinical research ethics committee of Hospital Clínico San Carlos de Madrid (Madrid, Spain) (code number 20/030-R_P). It was conducted in accordance with the Declaration of Helsinki.

The inclusion criteria for all patients consisted of being over 50 years old with healthy eyes, lens power within the available range from +10.00 to +30.00 D, regular corneal astigmatism of less than 1.0 D, and clear intraocular media, except cataracts. Exclusion criteria included previous ocular pathologies such as uveitis, age-related macular degeneration, or previous intraocular or corneal surgery; irregular astigmatism; and the presence of pupil abnormalities.

Monocular visual acuities and monocular contrast sensitivities were measured using the Clinical Trial Suite (CTS) device (M&S Technologies). Clinical data evaluated in this study correspond to measurements taken three months after surgery. All patients enrolled in the study underwent bilateral symmetric IOL implantation, but only their right eyes were evaluated.

Twenty-four participants were recruited between June 2020 and October 2022 for each group after calculating the sample size. To calculate the sample size, a pilot study was conducted with 10 patients, considering distance-corrected intermediate visual acuity (DCIVA) as the main variable. The mean value obtained was 0.26 ± 0.10 logMAR. Based on this standard deviation, an alpha risk of 0.05, a beta risk of 0.2 in a one-sided test, and an anticipated dropout rate of 10%, 24 subjects per group were required to recognize a statistically significant difference greater than or equal to 0.06 logMAR units.

All surgeries were performed under topical anesthesia. A 2.2 mm corneal incision and a paracentesis were made with a surgical knife. Anterior capsulotomy and nuclear fragmentation were carried out using a femtosecond laser with optical coherence tomography image control (CATALYS Precision System, Abbott Medical Optics, Inc., Irvine, CA, USA), while lens phacoemulsification utilized a commercial microsurgical system (Centurion Vision System, Alcon Laboratories, Inc., Geneva, Switzerland). Throughout the entire procedure, two ophthalmic viscosurgical devices were employed: cohesive sodium hyaluronate 1.0% (Healon, Johnson & Johnson, Santa Ana, CA, USA) and dispersive sodium hyaluronate 1.2% (Amvisc, Bausch & Lomb, Inc., Rochester, NY, USA). Subsequently, Isopure or Micropure IOLs were implanted into the capsular bag using a single-use injection system (123 system, Physiol, Liége, Belgium). The surgeries were supported by an assisted cataract surgery system (CALLISTO Eye from the Cataract Suite Markerless, Carl Zeiss Meditec AG). Upon completion of the procedures, patients received a combination of antibiotics, corticosteroids, and anti-inflammatory eye drops (moxifloxacin, dexamethasone, and bromfenac). All lens powers were calculated using the Barrett Universal II Formula, considering a lens factor (LF) of 2.09 to achieve emmetropia.

### 2.4. Data Analysis

The NIMO device generates a csv-format file for each measurement, which was loaded into an R script programmed to directly obtain power and aberrometric profiles. Statistical analysis and visualization were performed using Rstudio with R version 4.3.1 [14] and the graphic package ggplot2 [15]. Correlations between variables were studied by means of the Pearson’s correlation coefficient. Differences between groups (ISOPure vs. MicroPure) were tested using the unpaired *t*-test. The significance level to reach statistical significance was set to 5% (α = 0.05).

For the clinical part of the study, only one eye per subject was analyzed. To avoid bias, only data from the right eye were selected [16]. Thus, aside from avoiding possible biases, the correlation between optical bench results (obtained from a single lens) and clinical results makes more sense.

## 3. Results

### 3.1. Optical Bench

**Figure 1:**
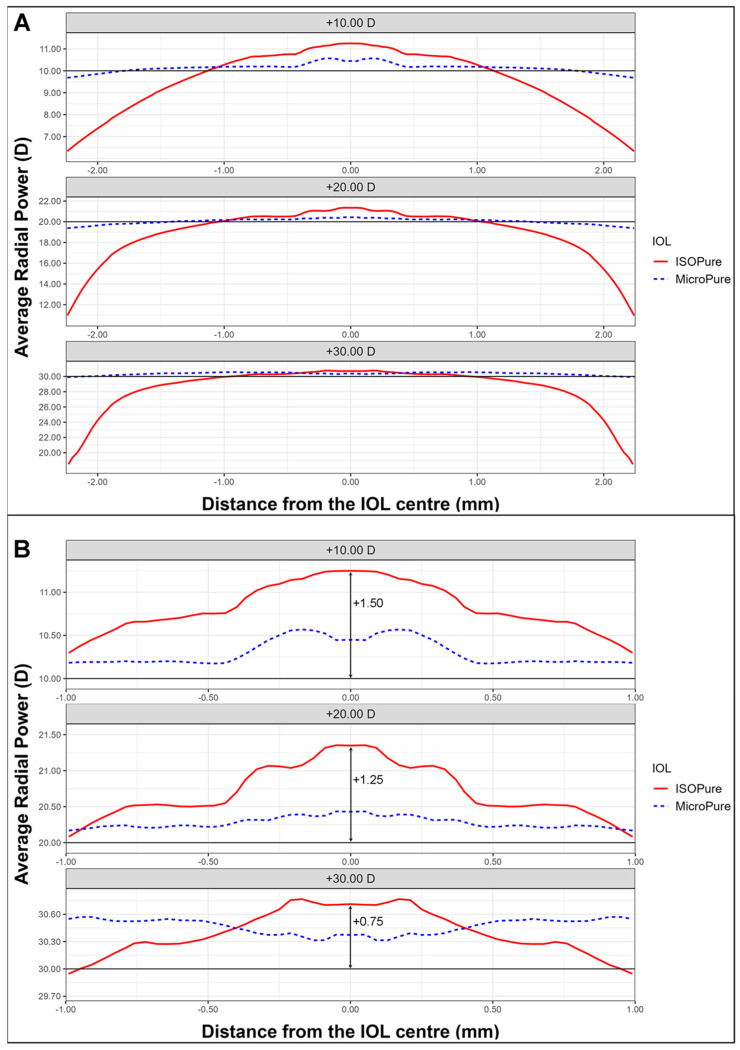
Average radial power maps for the three lens powers for the ISOPure (solid red line) and the MicroPure lenses (dashed blue line). (**A**) shows the values from the center to periphery, while (**B**) shows the data in the 2 central millimeters.

We will first present the power profiles as measured with NIMO. Figure 1A shows the power profiles, averaged through the whole surface, of an ISOPure lens (solid red line) and a MicroPure one (dashed blue line) with powers +10 D (upper), +20 D (middle), and +30 D (lower). Figure 1B shows an enlarged image of the central 2 mm diameter zone, allowing for clearer observation of the changes in central power for the two lens models.

The addition for the isofocal IOL depends on the nominal power. ISOPure lenses clearly present higher power than the standard monofocal lens due to the greater asphericity of their design. Indeed, if this power increment is considered as an addition, the lens with +10 D power presents approximately +1.50 D of addition at the center, and the respective values of addition for the +20 D and +30 D IOLs would be approximately +1.25 D and +0.75 D, respectively, as can be seen from Figure 1B.

**Figure 2:**
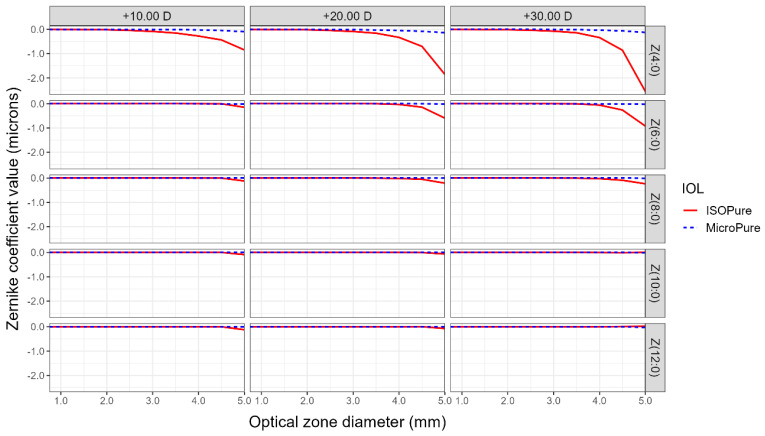
Zernike coefficients describing spherical aberrations of orders 4 to 12 according to optical zone size for the two lenses evaluated and the three nominal powers for the ISOPure (solid red line) and the MicroPure lenses (dashed blue line).

In addition to power profiles, NIMO can measure the wavefront aberrations of the lenses expressed, as usual, as a Zernike polynomial expansion. This allows us to study spherical aberration through analyzing the coefficients of the radially symmetrical Zernike polynomials of the 4th to 12th order. Figure 2 shows the coefficients of the Zernike polynomials for isofocal and monofocal standard lenses, for the three analyzed powers of +10.00, +20.00, and +30.00 D, and for different pupil diameters. The aspheric monofocal lens demonstrated a negative SA, closely resembling the laboratory-reported value of −0.11 microns for the coefficient Z(4,0). Notably, the isofocal lens is anticipated to display a significantly higher negative SA within the same area. Particularly, for a 5 mm diameter pupil, the Z(4,0) coefficient reaches values of −1.0, −2,0, and −2.5 microns, for the +10, +20, and +30 D power lenses, respectively. Notice that the negative SA of the isofocal lens is considerably reduced for all powers, when the pupil is lower than 3.5 mm. The sixth- and eighth-order SA also show clear differences when compared to the standard monofocal model.

### 3.2. Clinical Study

Table 1 shows the demographic data for patients included in the study.

Table 2 shows the visual acuities obtained for the monofocal aspheric and the isofocal lenses under photopic and mesopic conditions for far and intermediate distances. Differences in visual acuity were observed for intermediate vision at both 80 cm and 66 cm, with far distance correction, under photopic conditions. No differences were found under mesopic conditions, although visual acuity was slightly better at 66 cm for the ISOPure lens and similar between the two lenses at other distances.

When correlations were assessed using Pearson’s linear correlation coefficient between visual acuity values at different distances and pupil size, values of r < 0.3 were found, showing little effect.

Table 3 shows the correlations found when evaluating VAs obtained at different distances with pupil size.

**Figure 3:**
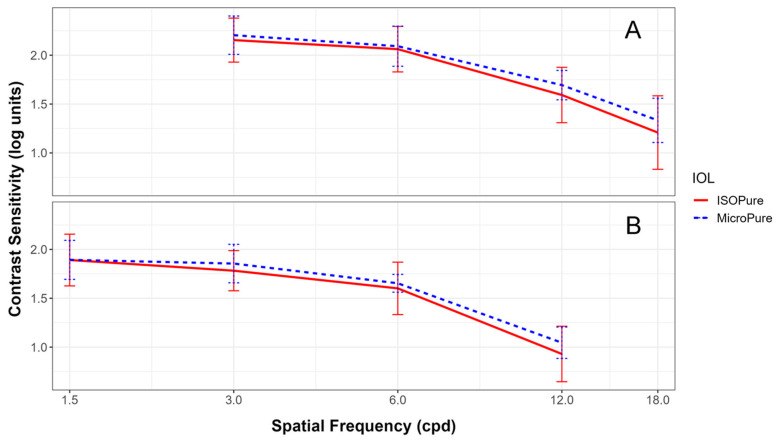
Monocular contrast sensitivities recorded in the Clinical Trial Suite (CTS) device at different spatial frequencies under photopic (3, 6, 12, 18 cpd) (**A**) and mesopic conditions (1.5, 3, 6, 12 cpd) (**B**) for the ISOPure (solid red line) and MicroPure lenses (dashed blue line).

Figure 3 shows the contrast sensitivity for both lenses under both photopic (Figure 3A) and mesopic conditions (Figure 3B).

As can be seen from Figure 3, contrast sensitivities were very similar for both lenses, under both lighting conditions, with no statistically significant differences at any spatial frequency tested. Photopic contrast sensitivities of both lenses were close to 2.17 for 3 cpd, 2.08 for 6 cpd, and 1.63 for 12 cpd. In mesopic conditions, the contrast sensitivities were close to 1.81 for 3 cpd, 1.63 for 6 cpd, and 1.00 for 12 cpd.

## 4. Discussion

With the introduction of new multifocal lens designs in the market, it is crucial to enhance our technical and clinical understanding of each lens to provide more precise prescriptions for patients. This knowledge becomes even more important when dealing with lenses that are not part of an existing product family or if they have a unique description provided by the manufacturer, such as the ISOPure lens, which is marketed as an isofocal model based on polynomial technology.

To the best of our knowledge, this study represents the first comparison between standard monofocal and isofocal lenses made of the same material and platform. We have conducted a comprehensive evaluation, examining both optical bench results and clinical outcomes, with a focus on establishing correlations between the obtained results.

### 4.1. Optical Bench

Regarding the observed radial powers, it was noted that the radial powers of the standard monofocal IOLs at +10.00 D, +20.00 D, and +30.00 D were very close to their nominal values (compare the dotted lines for the MicroPure IOL and the solid lines for the nominal powers in Figure 1). However, for the isofocal model, an elevated radial power was observed in the central zone, with a depression towards the periphery. This additional power varied depending on the power of the lens, resulting in approximate maximum additions of 1.50 D for +10.00 D, 1.25 for +20.00 D, and 0.75 for +30.00 D, in comparison to their nominal values.

Starting from an approximate optical zone of 2 mm, the power of the isofocal lens aligns with that of the standard monofocal lens but gradually decreases towards the periphery, coinciding with a significant increase in SA.

Spherical aberration, by definition, refers to the variation in the effective power of an optical system from the center to the periphery, which remains constant at any meridian of the lens. In the context of the ISOPure lens, as described by its designers, spherical aberration plays a crucial role in enabling patients to achieve improved visual acuity at intermediate and near distances compared to the standard monofocal model. Typically, primary spherical aberration, represented by the Zernike polynomial Z(4,0), is associated with spherical aberration. However, for the ISOPure lens, higher-order spherical aberrations also have significant implications. Through the analysis conducted on the optical bench, it is evident that the ISOPure lens exhibits notable contributions from Z(4,0), Z(6,0), and Z(8,0) polynomials, while the contributions from Z(10,0) and Z(12,0) orders are relatively less significant. In contrast, the MicroPure lens primarily demonstrates relevant values for the primary spherical aberration, with an approximate value of −0.11 µm for 4.75 mm for a lens of +20.00 D. However, for the ISOPure lens, an increase in spherical aberration is observed in the periphery starting at 4 mm.

Marcos [17], one of the ISOPure lens designers, presented the SA results for both lenses with a nominal power of +22.00 D within a 3 mm zone. The results revealed that the Micropure lens exhibited an SA of approximately 0.02 microns, whereas the ISOPure lens demonstrated an SA of −0.07 microns at that specific point, considering a cornea ISO2 with an SA of −0.27 µm. These findings closely align with those obtained using the NIMO device within the corresponding region.

### 4.2. Clinical Study

Our results indicate that, in terms of distance vision, the visual acuity (VA) achieved by patients with both lenses is similar. However, in intermediate vision at distances of 80 cm and 66 cm, the ISOPure lens demonstrates superior VA under photopic conditions. In mesopic conditions, the VA provided by the ISOPure lens remains comparable to the standard monofocal lens, without exhibiting the expected decline based on the optical bench results from 4 mm.

A study conducted by Stodulka et al. [18] examined the outcomes of the ISOPure lens, both monocularly and binocularly, and our findings align with their results obtained 4–6 months post intervention at the three evaluated distances. Additionally, our results are like those reported by Bova et al. [6], who assessed distance vision and the 66 cm intermediate distance and are also consistent with the far vision outcomes reported by Bernabeu-Arias [19], although comparisons cannot be made for other distances due to the binocular nature of Bernabeu-Arias’ study.

Regarding contrast sensitivity, Bova’s [6] results are comparable to ours, although their data were taken under binocular conditions 12 months after implantation, whereas our data are monocular and obtained 3 months after surgery. No other studies were found that evaluated contrast sensitivity under similar conditions to ours.

Mencucci et al. [20] compared the ISOPure IOL to two others enhanced monofocal models: Eyhance (Johnson & Johnson Vision Care, Inc., Jacksonville, FL, USA), and Vivinex (Hoya Surgical Optics, Singapore). While their results are not directly comparable to ours (as we compared ISOPure to a standard monofocal, not to other enhanced monofocals), the mean VA values obtained by Mencucci et al. are very similar to ours. They reported a LogMAR monocular UCVA of 0.04 ± 0.05 for the ISOPure IOL in their study, compared to 0.05 ± 0.13 in our study. Additionally, they found a LogMAR monocular DCVA of 0.02 ± 0.04 for the ISOPure IOL, while in our study, it was −0.01 ± 0.08. Our contrast sensitivity results are not directly comparable to those of Mencucci et al. since we measured CS monocularly, while they conducted their measurements binocularly.

Alarcon et al. [7] conducted an evaluation of the effect of pupil size on the through-focus optical performance of enhanced monofocal IOLs, including the ISOPure lens. Their results, obtained from optical bench measurements, demonstrated that the ISOPure lens provided performance like that of a spherical monofocal IOL, with a strong dependence on pupil size for far and intermediate vision. These findings align with our own observations on the optical bench, which revealed a distinct increase in addition in the central zone, accompanied by a reduction in power in the periphery and a significant increase in SA in the periphery. These factors may lead to poorer vision and visual quality for patients with larger pupils. However, these results do not align with clinical observations in patients. We found no relationship between visual acuity obtained at different distances and pupil size, and no significant differences were observed under both photopic and mesopic conditions. It is worth noting that pupil sizes are typically larger under mesopic conditions. These clinical findings were consistent when comparing the ISOPure lens to the standard monofocal lens.

A similar discrepancy in results between the optical bench and clinical data was also observed when evaluating the halo effect produced by the ISOPure lens compared to the MicroPure lens. Azor et al. [21] assessed the modulation transfer function (MTF) and halo observed on the optical bench for the ISOPure lens. They discovered that MTF values deteriorated significantly with 4.5 mm pupils compared to the values observed with 2 mm and 3.5 mm pupils, and the halo size also substantially increased. However, in a clinical study presented by Poyales et al. [22], no significant differences in either MTF values or patient-reported halo size values were found between a group of patients implanted with ISOPure and MicroPure lenses.

Our hypothesis suggests that the lack of correlation between the values obtained on the optical bench and the clinical values may be attributed to the Stiles–Crawford effect [23,24]. This effect is responsible for the perception of increased brightness when a light beam passes through the central part of the human eye’s pupil compared to passing through the pupil’s edge, even when covering an equal area. Consequently, light passing through the periphery of the pupil is less effective at stimulating vision compared to light passing near the center. This phenomenon could explain why the degradation observed in values measured on the optical bench for large pupils does not have the same impact in real-world conditions.

Furthermore, it is important to highlight the significance of the neuroadaptation process following intraocular lens implantation, as documented in the literature [25,26,27,28]. This process likely contributes to the observed differences between objective data and subjective clinical data.

One limitation of our study is the variation in preoperative pupil sizes among our patients, which was beyond our control due to the randomized nature of the study and the randomized assignment of the lens using a software. Consequently, it would be valuable to increase the sample size to include patients with larger pupils. To investigate whether the observed discrepancies between objective and subjective data persist, further investigation is needed.

## 5. Conclusions

After analyzing the results obtained in this study, we can conclude that the average radial power of an isofocal lens decreases from the center to the periphery of the lens, exhibiting the maximum addition in the central zone (2 mm) compared to the nominal value. In contrast, the standard monofocal model shows a stable mean radial power across the entire diameter. The isofocal lens shows a significantly higher negative SA for Z(4,0) compared to monofocal one, with the sixth- and eighth-order SA also showing clear differences when compared to the standard monofocal model.

Clinically, at far distances, the two lenses show similar results, while the ISOPure lens demonstrates better visual outcomes at the intermediate vision compared to the MicroPure lens under both photopic and mesopic conditions. Moreover, the improved performance of the isofocal lens remains unaffected by pupil enlargement, contrary to what the data obtained on the optical bench for large optical zones may suggest.

## Figures and Tables

**Table 1:** Preoperative demographic data for the two groups evaluated.

	ISOPure (*n* = 24)	MicroPure (*n* = 24)	*p*-Value
Gender (male/female; %)	41.67/58.33	45.84/54.16	
Age (mean ± SD) (years)	71.71 ± 6.13 (range: 62 to 84)	71.90 ± 6.45 (range: 61 to 88)	0.824
IOL power (D)	22.00 ± 2.32 (range: 18.5 to 26.50)	21.25 ± 2.10 (range: 16.00 to 24.00)	0.351
Pupil photopic (mm)	3.00 ± 0.46 (range: 2.34 to 4.13)	3.41 ± 0.65 (range: 2.41 to 4.43)	0.006 *
Pupil mesopic (mm)	4.13 ± 0.53 (range: 3.10 to 5.41)	4.49 ± 0.83 (range: 3.26 to 5.86)	<0.001 *

* Statistically significant difference.

**Table 2:** Postoperative monocular logMAR visual acuities at different distances under photopic and mesopic luminance conditions in experimental (ISOPure) and control (MicroPure) groups. Mean ± SD (range). UDVA: uncorrected distance visual acuity; CDVA: corrected distance visual acuity; DCIVA: distance-corrected intermediate visual acuity.

MONOCULAR VISUAL ACUITIES (logMAR Units ± SD)						
	Photopic (85 cd/m^2^)			Mesopic (3.5 cd/m^2^)		
	ISOPure (*n* = 24)	MicroPure (*n* = 24)	*p*-Value	ISOPure (*n* = 24)	MicroPure (*n* = 24)	*p*-Value
UDVA (4 m)	0.05 ± 0.13 (−0.12–0.32)	0.01 ± 0.07 (−0.14–0.14)	0.181	0.17 ± 0.12 (−0.04–0.48)	0.14 ± 0.09 (−0.08–0.28)	0.495
CDVA (4 m)	−0.01 ± 0.08 (−0.12–0.18)	−0.03 ± 0.06 (−0.16–0.06)	0.350	0.11 ± 0.09 (−0.06–0.40)	0.08 ± 0.08 (−0.10–0.24)	0.395
DCIVA (80 cm)	0.18 ± 0.10 (−0.02–0.54)	0.24 ± 0.09 (0.10–0.42)	0.014 *	0.37 ± 0.10 (0.20 –0.60)	0.36 ± 0.09 (0.14–0.50)	0.634
DCIVA (66 cm)	0.23 ± 0.11 (0.06–0.52)	0.30 ± 0.12 (0.12–0.54)	0.022 *	0.42 ± 0.11 (0.24–0.66)	0.43 ± 0.10 (0.20–0.58)	0.975

* Statistically significant difference.

**Table 3:** Pearson correlation coefficient values (and statistical significances in parentheses) for visual acuities and pupil size. CDVA: corrected distance visual acuity; DCIVA: distance-corrected intermediate visual acuity.

	Pearson Correlation Coefficients (*p*-Value)	
	ISOPure	MicroPure
CDVA—Photopic pupil	0.138 (0.521)	0.348 (0.096)
DCIVA (80 cm)—Photopic Pupil	−0.047 (0.826)	0.387 (0.062)
DCIVA (66 cm)—Photopic Pupil	0.112 (0.603)	0.442 (0.031)
CDVA—Mesopic pupil	0.119 (0.581)	0.389 (0.06)
DCIVA (80 cm)—Mesopic Pupil	0.039 (0.858)	0.255 (0.229)
DCIVA (66 cm)—Mesopic Pupil	0.200 (0.348)	0.334 (0.111)

## Data Availability

Due to privacy concerns, all the data of this study are kept private.

## References

[1] Fernandez J., Rocha-de-Lossada C., Zamorano-Martin F., Rodriguez-Calvo-de-Mora M., Rodriguez-Vallejo M. (2023). Positioning of enhanced monofocal intraocular lenses between conventional monofocal and extended depth of focus lenses: A scoping review. BMC Ophthalmol..

[2] Rampat R., Gatinel D. (2021). Multifocal and Extended Depth-of-Focus Intraocular Lenses in 2020. Ophthalmology.

[3] Megiddo-Barnir E., Alio J.L. (2023). Latest Development in Extended Depth-of-Focus Intraocular Lenses: An Update. Asia Pac. J. Ophthalmol..

[4] Fernández Gutiérrez D., Barbero Briones S., Dorronso Díaz C., Marcos Celestino S. (2013). Refractive Multifocal Intraocular Lens with Optimised Optical Quality in a Range of Focus and Method to Produce It. European Patent.

[5] Alonso J., Gómez-Pedrero J.A., Quiroga J.A. (2019). Modern Ophthalmic Optics.

[6] Bova A., Vita S. (2022). Clinical and Aberrometric Evaluation of a New Monofocal IOL with Intermediate Vision Improvement. J. Ophthalmol..

[7] Alarcon A., Canovas C., Koopman B., Pande M.V., Koch D.D., Piers P. (2023). Optical bench evaluation of the effect of pupil size in new generation monofocal intraocular lenses. BMC Ophthalmol..

[8] Joannes L., Dubois F., Legros J.-C. (2003). Phase-shifting Schlieren: High-resolution quantitative schlieren that uses the phase-shifting technique principle. Appl. Opt..

[9] de Groot P., Leach R. (2011). Phase Shifting Interferometry. Optical Measurements of Surface Topography.

[10] Joannes L., Jacot M., Hutsebaut X., Dubois X. (2019). NIMO TR1504 Software User Guide.

[11] Vargas J., Gómez-Pedrero J.A., Alonso J., Quiroga J.A. (2010). Deflectometric method for the measurement of user power for ophthalmic lenses. Appl. Opt..

[12] Wade N., Swanston M. (2012). Visual Perception: An Introduction.

[13] Gómez-Pedrero J., Albarrán C., Garcia-Montero M., Garzón N., Fernández-González V. (2023). Influence of instrumental factors in the measurement of power profiles of intraocular lenses with a commercial deflectometer. Appl. Sci..

[14] R Core Team (2022). R: A Language and Environment for Statistical Computing.

[15] Wickham H. (2010). A Layered Grammar of Graphics. J. Comput. Graph. Stat..

[16] Armstrong R.A. (2013). Statistical guidelines for the analysis of data obtained from one or both eyes. Ophthalmic Physiol. Opt..

[17] Marcos S. Isofocal IOL Concept. Proceedings of the ISOP Presbyopia.

[18] Stodulka P., Slovak M. (2021). Visual Performance of a Polynomial Extended Depth of Focus Intraocular Lens. Open J. Ophthalmol..

[19] Bernabeu-Arias G., Beckers S., Rincón-Rosales J.L., Tañá-Rivero P., Bilbao-Calabuig R. (2023). Visual Performance at Different Distances After Implantation of an Isofocal Optic Design Intraocular Lens. J. Refract. Surg..

[20] Mencucci R., Morelli A., Cennamo M., Roszkowska A.M., Favuzza E. (2023). Enhanced Monofocal Intraocular Lenses: A Retrospective, Comparative Study between Three Different Models. J. Clin. Med..

[21] Azor J.A., Vega F., Armengol J., Millan M.S. (2022). Optical Assessment and Expected Visual Quality of Four Extended Range of Vision Intraocular Lenses. J. Refract. Surg..

[22] Poyales F., Charbel C., Rico L., Pérez L., Garzon N. (2021). Visual performance of isofocal extended depth-of-focus lens versus standard monofocal intraocular lens. Visual Performance of Isofocal Extended Depth-of-Focus Lens versus Standard Monofocal Intraocular Lens.

[23] Stiles W.S., Crawford B.H. (1933). The luminous efficiency of rays entering the eye pupil as different points. Proc. R. Soc. B.

[24] Westheimer G. (2008). Directional sensitivity of the retina: 75 years of Stiles-Crawford effect. Proc. R. Soc. B.

[25] Zhang L., Lin D., Wang Y., Chen W., Xiao W., Xiang Y., Zhu Y., Chen C., Dong X., Liu Y. (2021). Comparison of Visual Neuroadaptations After Multifocal and Monofocal Intraocular Lens Implantation. Front. Neurosci..

[26] Rosa A.M., Miranda A.C., Patricio M.M., McAlinden C., Silva F.L., Castelo-Branco M., Murta J.N. (2017). Functional magnetic resonance imaging to assess neuroadaptation to multifocal intraocular lenses. J. Cataract. Refract. Surg..

[27] Fernandes P., Ferreira C., Domingues J., Amorim-de-Sousa A., Faria-Ribeiro M., Queirós A., González-Meijome J.M. (2022). Short-term delay in neural response with multifocal contact lens might start at the retinal level. Doc. Ophthalmol..

[28] Rosa A.M., Miranda A.C., Patricio M., McAlinden C., Silva F.L., Murta J.N., Castelo-Branco M. (2017). Functional Magnetic Resonance Imaging to Assess the Neurobehavioral Impact of Dysphotopsia with Multifocal Intraocular Lenses. Ophthalmology.

